# 2024 List of Reviewers

**DOI:** 10.1117/1.NPh.12.1.010102

**Published:** 2025-01-16

## Abstract

Thanks to reviewers who served Neurophotonics in 2024.

*Neurophotonics* would like to sincerely thank the following individuals who served as reviewers in 2024. The success of our publication hinges on the voluntary contributions of time and energy put forth by these professionals.

Ardalan Aarabi
Androu Abdalmalak
Lamiae Abdeladim
Nicolò Accanto
Sung Ji Ahn
Anna Letizia Allegra Mascaro
Mahnoush Amiri
Lyubov Amitonova
Pascal Auzou
Christopher Baker
Wesley Baker
Lisa Bastian
Ken Berglund
Giulio Bicciato
Addison Billing
Pablo Blinder
Augusto Bonilauri
Oliver Bracko
Sabrina Brigadoi
Craig Brown
Chiara Bulgarelli
Fabio Carbone
Guo Chen
Siyu Chen
Tom Cheng
Xiaojun Cheng
Antonio Chiarelli
Sangcheon Choi
Julian Clarke
Danut Adrian Cojoc
Robert Cooper
Joao Couto
Hamid Dehghani
Michele Desjardins
Rick Dijkhuizen
Pu-ting Dong
Viktor Dremin
Adam Eggebrecht
Daniel Elson
Emilia Entcheva
Sefik Evren Erdener
G. Bard Ermentrout
Dan Fu
Jessica Gemignani
Judit Gervain
Emily Gibson
Ethan Goldberg
Manuel Gomez-Ramirez
Edgar Guevara
Martin Haesemeyer
Yun He
Ingo Helmich
David Highton
Roger Ho Chun Man
Yoko Hoshi
Hyacinth Hyacinth
Krishna Inavalli
Sagi Jaffe-Dax
Jingjing Jiang
Chulmin Joo
Dongkyun Kang
Rini Kaplan
Deepa Kasaragod
Qutaibeh Katatbeh
Hiroshi Kawaguchi
Kivilcim Kilic
Beop-Min Kim
MinWoo Kim
Yongsoo Kim
Franziska Klein
Alexxai Kravitz
Jasmine Kwasa
Marcel Lauterbach
Jérôme Lecoq
Kijoon Lee
Seung Yup Lee
Frédéric Lesage
Jennifer Li
Jiawen Li
Lei Li
Mingsheng Li
Rihui Li
Chang Liu
Chao Liu
Quan Liu
Gaia Lucarini
Shangbang Luo
Pierre Mahou
Giovani Martins
Niall McAlinden
Rickson Mesquita
Daniel Milej
Miguel Mireles Nuñez
Aseema Mohanty
Samuel Montero-Hernandez
Philip Morgan
Amreen Mughal
Timothy Murphy
Laleh Najafizadeh
Thien Nguyen
Nozomi Nishimura
Ilkka Nissila
Stephanie Nolen
Sergio Novi Junior
Justin O’Hare
Umberto Olcese
Cees Otto
Ishara Paranawithana
Boris Park
Vicente Parot
Andre Paschoal
Martin Paukert
Yannis Paulus
Stephen Payne
Darcy Peterka
Eric Petersen
Martin Ploschner
Kaspar Podgorski
Manojit Pramanik
Sean Quirin
Patrick Reeson
Mitchell Robinson
Drew Robson
Ravi Rungta
Peter Rupprecht
Shuzo Sakata
Tilmann Sander-Thömmes
Hendrik Santosa
Felix Scholkmann
Julia Scott
Derek Sederman
Ikbal Şencan-Eğilmez
Myeongsu Seong
Kristen Severi
Arefeh Sherafati
Ruth Sims
Spencer Smith
Taeyoon Son
Keith St. Lawrence
Wan-chun Su
Jinyan Sun
Ulas Sunar
John Sunwoo
Yaroslav Sych
Jianbo Tang
Lei Tian
Jan Tonnesen
Maria Leilani Torres-Mapa
Alessandro Torricelli
Cam Ha Tran
Jason Trobaugh
Vassiliy Tsytsarev
Kevin Turner
Tara Urner
Matthieu Vanni
Alberto Vazquez
Ernesto Vidal Rosas
Fabian Voigt
Jakob Voigts
Alexander Von Luehmann
Ping Wang
Tianyu Wang
Sossena Wood
Kuan Cheng Wu
Edward Xu
Vladislav Yakovlev
Shijie Yan
Chen Yang
Mohammad Yaseen
Guoqiang Yu
Zhen Yuan
Meryem Yücel
Mark Zarella
Qingguang Zhang
Wei-Ting Zhang
Xian Zhang
Yide Zhang
Hubin Zhao

